# Joint Contracture Resulting From a Two-Hour Daily Joint Immobility: An Experimental Study in Rats

**DOI:** 10.7759/cureus.112188

**Published:** 2026-07-06

**Authors:** Senma Nagi, Masahiro Sugahara, Ryoma Ikeo, Kazuya Sakai, Yuta Sato, Takeya Ono

**Affiliations:** 1 Biological System Sciences, Prefectural University of Hiroshima, Mihara, JPN; 2 Rehabilitation Science, Osaka Health Science University, Osaka, JPN; 3 Rehabilitation, Yara Orthopedic Clinic, Fukuoka, JPN; 4 Physical Therapy, Prefectural University of Hiroshima, Mihara, JPN

**Keywords:** hindlimb suspension, joint contracture, joint immobility, joint range of motion, lack of joint movement

## Abstract

Background

Although joint contracture prevention has mainly focused on exercise time, an effective method has not yet been established. A lack of joint movement is considered to be associated with the development of joint contracture; however, this relationship has not been quantitatively investigated. As repositioning is typically performed every two hours, intensive care unit patients and bedridden older adults may experience at least two consecutive hours of immobility.

Objective

This study investigated whether joint contractures develop after two hours of joint immobility per day, over two weeks, in a non-weight-bearing state, due to hindlimb suspension.

Methods

The experimental animals were six eight-week-old male Wistar rats weighing 349-380 g (average: 363.8 ± 13.5 g). In the immobilization group, the right hindlimbs of all six rats were immobilized in a maximum plantar flexion position, in which the toes were directed downward, whereas the left hindlimbs of the same six rats served as the control group, with joint movement not restricted. The measured items included the range of joint motion (ankle dorsiflexion angle), muscle fiber cross-sectional area, and soleus muscle relative weight ratio.

Results

The range of joint motion differed significantly between these groups after two weeks, and the immobilization group showed significant changes between the start of the experiment and after two weeks. The ankle dorsiflexion angle decreased by 1.7% in the control group and by 9.8% in the immobilization group over two weeks.

Conclusions

These findings suggest that two hours of daily joint immobility combined with hindlimb suspension was associated with a reduction in ankle dorsiflexion range of motion and may contribute to contracture development. This model provides a new perspective that differs from conventional experimental models using continuous joint immobilization during the experimental period.

## Introduction

The development of joint contracture can restrict the performance of basic movements, such as standing up and walking, which are important for leading a normal life, and has a significant negative impact on activities of daily living [1,2]. Research on joint contracture has mainly used animal experiments. For example, according to Williams [3], preventing joint contracture requires avoiding joint immobilization for 30 min daily and encouraging joint movement. Based on these results, research has been carried out to prevent the occurrence of joint contracture in humans. Harvey et al. [4] reported that, although they carried out joint movement for 30 min daily on the ankle joints of patients with spinal cord injuries, it was not effective in preventing the occurrence of joint contracture. In addition, Horsley et al. [5] reported that 30 min of stretching per day was ineffective in preventing joint contracture in the wrists of patients with cerebrovascular disease. Past research has mainly focused on the amount of time spent exercising the joints; however, the results have not necessarily indicated effective prevention methods, and further research is required.

Wagner et al. [6] reported that approximately two-thirds of older adult care facility residents had one or more joint contractures. Tariq et al. [7] also summarized a review of the factors causing joint contractures. These studies suggest that pain, muscle weakness, reduced physical activity, bed rest, and other factors that reduce joint movement are associated with the development of joint contractures. However, these studies neither actually monitored nor recorded joint movement, nor did they quantitatively examine whether a lack of joint movement was the cause of joint contracture. Therefore, further clarification of the relationship between the two is necessary.

Trudel et al. [8] stated that joint contracture occurs due to joint fixation and that the longer the period of joint fixation, the greater the restriction of the range of motion (ROM). Consequently, joint fixation is thought to be the main cause of joint contracture. In considering the prevention and improvement of joint contracture, we thought it would be useful to focus on the “immobility time of the joint” rather than the “movement time of the joint.” Therefore, we defined the lack of joint movement as “repeated short periods of daily joint immobilization,” and then examined its relationship with the occurrence of joint contracture.

Many animal experiments on joint contracture have been performed using continuous joint immobilization. In these, plantar flexion immobilization is common in the ankle joint [3,9,10]. Although the lower limbs of humans in bed are non-weight-bearing, Morey-Holton and Globus [11] reported a hindlimb suspension model that reproduced the non-weight-bearing state of the lower limbs in rats. In addition, several studies have reported that patients and residents who are unable to turn over by themselves generally have their positions changed every two hours [12-15]. Based on these findings, we considered that patients in the intensive care unit (ICU) and bedridden older adults may be at risk of experiencing joint immobility for at least two hours continuously.

Therefore, in this study, we adopted an intermittent immobilization model, which differs from that by Trudel and Uhthoff [16], who immobilized the joints for 24 h daily during the experimental period. Instead, the current study immobilized the joints for just two hours, allowing free movement in the remaining time. Additionally, the current model was designed to mimic temporary joint immobilization and a non-weight-bearing state, which were achieved clinically by combining joint immobilization with hindlimb suspension. Furthermore, since Trudel and Uhthoff confirmed that joint contracture in rats occurred after two weeks of immobilization [16], we considered a two-week period as sufficient for observing the onset of contracture and set our experimental period to two weeks. We hypothesized that repeated two-hour daily ankle immobilization under hindlimb suspension would be associated with a reduction in ankle dorsiflexion ROM after two weeks. Therefore, this study aimed to investigate whether joint contracture occurs after two weeks of daily joint immobility for two hours in a non-weight-bearing state due to hindlimb suspension.

## Materials and methods

Animals and experimental groups

The experimental animals were six male Wistar rats aged eight weeks and weighing 349-380 g (average: 363.8 ± 13.5 g). The sample size was determined by considering the 3R principles of animal experimentation (Replacement, Reduction, and Refinement), particularly reduction of the number of animals used, and by referring to previous rat studies on joint contracture that used small sample sizes of five or six rats per group [9,10,17]. The right lower limbs of each rat were assigned to the immobilization group during the period of immobilization, whereas the left lower limbs were assigned to the control group. All rats were housed in a breeding cage (27 cm wide × 44 cm long × 18.7 cm high) with free access to water and food. The experimental period was two weeks. During the experiment, the temperature in the breeding room was maintained at 23°C. The light/dark cycle was controlled using an automatic lighting system and set to a 12-h cycle. For all rats, experiments were performed after confirming that the anesthesia was sufficiently effective and taking care not to cause pain. After inhalation anesthesia with isoflurane as the induction agent, a mixture of three anesthetics (Dormitor, midazolam, and Vetrufal) was administered intraperitoneally. This study was carried out using the animal experiment facility attached to our university and was approved by the Research Ethics Committee of our university (approval No. 21A-004-2).

Hindlimb suspension and joint fixation

**Figure 1:**
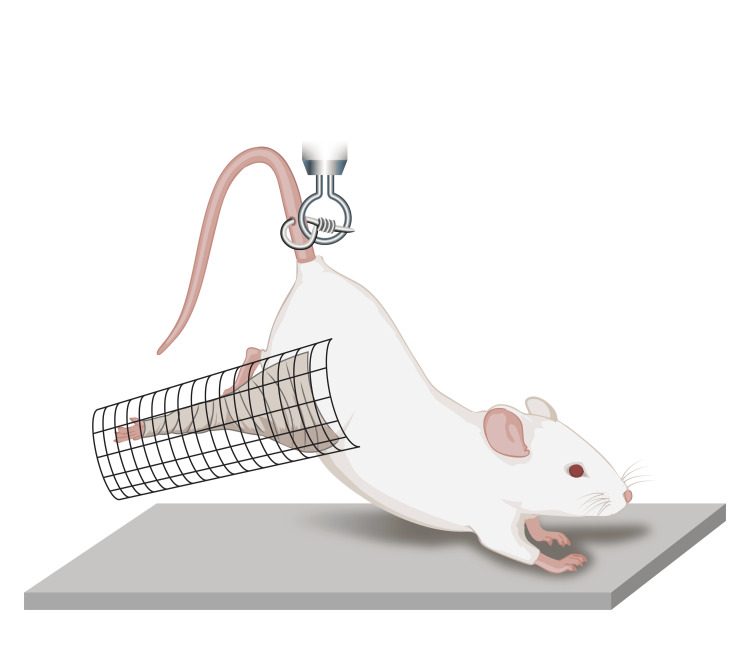
Animal model of hindlimb suspension A loop is formed by passing a 1.0-mm Kirschner wire through the rat vertebrae proximal to the tail in a horizontal direction, and a suspension site is created by passing the wire through a ring. The right lower limb is immobilized in the maximum plantar flexion position at the ankle joint. The thigh to the tip of the foot is fixed with a tape and protected with a wire mesh. The left lower limb is included as the control group, with no restriction of the joint movement. Image credit: Created by the authors using Adobe Illustrator (Adobe Inc., California, US).

Consistent with the method by Sato et al. [17], a 1.0-mm Kirschner wire was used to pierce the rat vertebrae near the tail horizontally to form a loop, and a suspension site was created by passing it through a ring (Figure 1).

**Figure 2:**
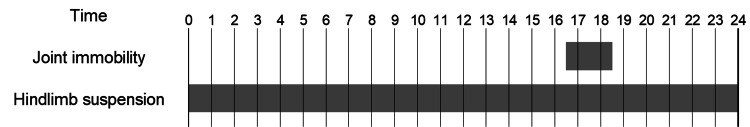
Daily schedule during the experimental period Gray boxes indicate the time of implementation.

The right lower limb was immobilized, with the ankle joint in the maximum plantar flexion position, in which the toes were directed downward, and immobilization was maintained for two hours daily from 16:30 to 18:30 (Figure 2).

The immobilized joint region extended from the thigh to the tip of the foot, and the toes were exposed to check for the development of edema. The joints were immobilized using taping and protected with wire mesh to prevent damage. Thus, the present experimental model combined hindlimb suspension with intermittent ankle immobilization for two hours per day.

Measurement of the joint angles

In all groups, the ROM in dorsiflexion of the ankle joint was measured under anesthesia before the start of the experiment. After the experiment, measurements were taken following euthanasia. Before the procedure, the rats were gently removed from their cages to minimize stress. Room temperature was maintained at 23°C throughout the peri-procedural period, including before anesthesia, during ROM measurement, and during recovery, to prevent hypothermia. Skin color, eye color, and respiratory pattern were monitored during the procedure to detect any abnormalities.

After the procedure, the rats were gently returned to their cages and placed in the supine position with attention to maintaining airway patency, and skin color, eye color, and respiratory pattern were continuously observed during recovery. The measurements were performed with the rats placed in a lateral lying position, and the hip and knee joints passively positioned at maximum flexion. In line with the method by Honda et al. [18], the ankle joint was dorsiflexed with a force of 0.3 N using a small force gauge (LTS-1KA, Kyowa Electronic Instruments Co., Ltd., Tokyo, Japan), and the ankle joint was photographed using a digital camera from a vertical overhead perspective. Using the images acquired, the angle of dorsiflexion of the ankle joint was measured using image analysis software (ImageJ ver. 1.54 g, National Institutes of Health (NIH), Maryland, USA). In this case, the basic axis was the line connecting the fibula and lateral malleolus, and the moving axis was the line parallel to the plantar surface of the calcaneus. The angle measurement indicated that a smaller value corresponded to a more restricted joint range of motion. The ankle dorsiflexion angle was measured once from each image by a single evaluator who was aware of the group and measurement time point. Repeated measurements and intraobserver or interobserver variability assessments were not performed.

Tissue sampling and histological analysis

After the experimental period, the body weight was measured, and the soleus muscles of the immobilized and control groups were removed and weighed to determine the muscle wet weight, which was then used for histological analysis. The extracted soleus muscles were rapidly frozen in isopentane cooled with dry ice, and 10 µm-thick transverse sections were prepared using a cryostat. The sections were then stained with hematoxylin-eosin (H&E) for morphological observation and examined under an optical microscope. The H&E-stained sections were photographed at 400x magnification using a microscope digital camera, and the images were sequentially imported into a computer. From the images acquired, the cross-sectional areas of >100 muscle fibers were measured per sample, and the average value was considered the measurement value [18]. The wet weight of the soleus muscle was divided by the body weight to obtain the relative weight ratio of the soleus muscle.

Statistical analysis

Statistical analyses were performed using R software version 4.6.0 (R Foundation for Statistical Computing, Vienna, Austria). For the joint ROM, a two-way repeated-measures analysis of variance was performed with the dependent variable being the angle of dorsiflexion of the ankle joint, and the independent variables being the time of measurement (the start of the experiment and after two weeks) and group (immobilization and control groups). To account for the paired contralateral-limb design and repeated measurements over time, each animal was treated as the subject factor in the analysis. Considering the interaction between the time of measurement and group, simple main effects were examined. A significance level of <5% was used to determine a statistical significance. The soleus muscle cross-sectional area and the soleus muscle relative weight ratio were compared between the immobilization and control groups using paired t-tests, because the immobilized and control limbs were obtained from the same animals. A significance level of <5% was used to determine statistical significance.

## Results

Range of motion

The results of the measurement of the dorsiflexion angle of the ankle joint at the start of the experiment and after two weeks are shown in Table 1.

**Table 1:** Ankle dorsiflexion angle at each measurement time point in each group (degrees) ^*^p<0.01 vs control group at the same time point, based on the simple main effect of group; ^†^p<0.001 vs the start of the experiment within the same group, based on the simple main effect of time. Values are presented as mean ± standard deviation. A two-way repeated-measures ANOVA was performed while accounting for the paired contralateral-limb design and repeated measurements over time. Significant effects were observed for group (F(1,5) = 11.68, p=0.019), time (F(1,5) = 21.52, p=0.006), and the interaction between group and time (F(1,5) = 16.97, p=0.009).

Group	At the start of the experiment	Two weeks later
Control group	136.1 ± 3.4	133.8 ± 6.2
Immobilization group	135.5 ± 4.3	122.2 ± 3.6^*†^

Statistical analysis revealed significant effects for group (F(1,5) = 11.68, p=0.019), time (F(1,5) = 21.52, p=0.006), and the interaction between group and time (F(1,5) = 16.97, p=0.009). Because the interaction between group and time was significant, simple main effects were examined. Although no significant difference was observed between the groups at the start of the experiment (t(5) = -0.39, p=0.714), a significant difference was observed after two weeks (t(5) = -4.11, p=0.009).

At two weeks, the mean difference between the immobilization and control groups was -11.60° (95% confidence interval or CI: -18.84 to -4.35°). In addition, a significant difference was observed between the start of the experiment and two weeks later in the immobilization group (t(5) = 7.71, p<0.001); however, no significant difference was observed between the start of the experiment and two weeks later in the control group (t(5) = 0.92, p=0.400). In the immobilization group, the mean decrease from the start of the experiment to two weeks later was 13.36° (95% CI: 8.91 to 17.82°). The percentage decrease in ankle dorsiflexion angle from the start of the experiment to two weeks later was 1.7% in the control group and 9.8% in the immobilization group.

Soleus muscle cross-sectional area

The results of the soleus muscle cross-sectional area measurements after two weeks are shown in Table 2.

**Table 2:** Comparison of muscle cross-sectional area and soleus muscle relative weight ratio after two weeks Values are presented as mean ± standard deviation. Comparisons between the control and immobilization groups were performed using paired t-tests because both limbs were obtained from the same animals.

Outcome	Control group	Immobilization group	t value (df)	p value
Muscle cross-sectional area (μm²)	881.3 ± 155.0	861.6 ± 277.7	t(5) = -0.18	0.865
Relative weight (mg/g)	0.22 ± 0.01	0.21 ± 0.02	t(5) = -0.12	0.906

The paired t-test showed no significant difference between the immobilization and control groups (t(5) = -0.18, p=0.865).

Soleus muscle relative weight ratio

The results of the soleus muscle relative weight ratio calculations after two weeks are shown in Table 2. The paired t-test showed no significant difference between the immobilization and control groups (t(5) = -0.12, p=0.906).

## Discussion

Range of motion

In this study, we set the immobilization time for the joints to two hours per day based on the finding that repositioning is generally carried out every two hours. As the results showed, the range of joint motion decreased. Even with immobilization for a short period of two hours per day, a reduction in ankle dorsiflexion ROM was observed. These findings suggest that repeated short periods of joint immobility may contribute to the development of joint contracture. Sato et al. [17] used a rat hindlimb suspension model to show that a non-weight-bearing state delays the recovery of joint contracture, and based on this result, it can be inferred that a non-weight-bearing state contributes to the worsening of joint contracture. In this study, the non-weight-bearing condition may also have contributed to the observed reduction in ankle dorsiflexion ROM.

In addition, according to Trudel and Uhthoff [16], the involvement rate of myogenic restrictive factors in joint contracture is highest two weeks after joint fixation, and subsequently, the involvement rate of arthrogenic restrictive factors increases. Based on this previous finding and the two-week experimental period used in the present study, myogenic restrictive factors may have contributed to the observed reduction in ankle dorsiflexion ROM. However, because the present study did not directly distinguish between myogenic and arthrogenic restrictive factors, this interpretation should be regarded as a hypothesis. The occurrence and progression of joint contracture is a type of inflammatory response, and it is thought that the fibrosis and scarring of tissues during the recovery period of inflammation cause a decrease in muscle flexibility, which in turn leads to a restriction of the joint ROM [19,20].

Cross-sectional area and relative weight of the soleus muscle

The cross-sectional area and relative weight of the soleus muscle showed no significant difference between the immobilization and control groups. Fujita et al. [21] reported that when the ankle joints of rats with hindlimb suspension models were continuously immobilized for two weeks, the wet weight and cross-sectional area of the soleus muscle in the plantar flexion immobilization group were significantly lower than those in the control group. However, in this study, the immobilization period was only two hours per day over the same two-week experimental period; therefore, the total immobilization time was very short. This short total immobilization time may explain the absence of significant differences in the soleus muscle cross-sectional area and soleus muscle relative weight ratio. However, given the small sample size, this finding should be interpreted cautiously, as subtle muscle involvement may not have been detected by the present outcome measures. Furthermore, measurements of soleus muscle cross-sectional area and relative weight ratio alone may not detect subtle muscular or intramuscular connective tissue changes, such as early fibrosis, changes in passive stiffness, or collagen remodeling.

Limitations and future directions

This study has some limitations. First, because no a priori sample size calculation was performed and only six rats were used, this study should be regarded as an exploratory pilot study. Therefore, the absence of significant differences in soleus muscle cross-sectional area and relative weight ratio should be interpreted cautiously, as it may reflect limited statistical power rather than definitive evidence that disuse-related changes in the soleus muscle did not occur. Second, because the present model combined hindlimb suspension with intermittent ankle immobilization, the effects of joint immobility and non-weight-bearing could not be separated. In addition, because no group without hindlimb suspension was included, it remains unclear whether the observed reduction in ankle dorsiflexion range of motion was specifically attributable to the two hour daily immobilization period alone. Third, the present study used a paired contralateral-limb design, with the right hindlimb assigned to the immobilization group and the left hindlimb assigned to the control group. Although this design reduces inter-animal variability, possible systemic effects of hindlimb suspension and interactions between the contralateral limbs cannot be completely excluded. Fourth, the ankle dorsiflexion angle was measured by a single non-blinded evaluator, and only one measurement was obtained from each image. Intraobserver and interobserver reliability were not assessed. Therefore, measurement bias and measurement variability cannot be excluded, which limits the reproducibility of the primary outcome. Fifth, the histological assessment in this study was limited to the measurement of soleus muscle fiber cross-sectional area. We did not evaluate tissue changes such as fibrosis, collagen deposition, inflammatory changes, or extracellular matrix remodeling in the muscle or articular/periarticular tissues, including the joint capsule, ligaments, and synovium. Therefore, the mechanisms underlying the observed reduction in ankle dorsiflexion ROM cannot be fully determined from the present findings, and further studies including histological assessment of muscle fibrosis and articular/periarticular tissues are needed. Sixth, the study was conducted in rats rather than humans. Although approximately 90% of human and rat genes are shared [22], notable physiological differences-such as lifespan, heart rate, and metabolic rate-exist between the species [23]. In addition, the present model does not directly reproduce the complex immobilization patterns observed in ICU patients or bedridden older adults. Therefore, caution is required when extrapolating these findings to human clinical populations. Seventh, the experimental period was limited to two weeks, making it unclear whether joint contracture could occur in a shorter timeframe, such as within one week. Clarifying this point would enhance the clinical applicability of the results. Eighth, the immobilization period was fixed at two hours per day; however, it remains uncertain whether this duration represents the minimum threshold for inducing contracture.

This experimental model was designed to reproduce one aspect of clinical immobility, namely repeated short periods of joint immobility under a non-weight-bearing condition, which may occur in ICU patients and bedridden older adults. However, actual immobilization patterns in these populations are more complex and variable, depending on factors such as posture, muscle tone, underlying disease, nursing care, and rehabilitation intervention. Therefore, this model should be interpreted as a preliminary and exploratory rat model for investigating intermittent joint immobility, rather than a direct reproduction of clinical immobilization patterns in humans. Future studies should test shorter immobilization times to determine this lower limit. Additionally, the development of contracture may vary depending on the interaction between immobilization duration and experimental period, which should be explored in subsequent investigations.

## Conclusions

This preliminary study showed that two hours of daily joint immobility combined with hindlimb suspension was associated with a reduction in ankle dorsiflexion ROM in a small rat model. These findings suggest that repeated short periods of joint immobility may contribute to contracture development, although a causal relationship cannot be determined from the present study alone. Because the present model combined intermittent ankle immobilization with hindlimb suspension and included only six rats, the findings should be interpreted as an association with the experimental condition rather than definitive evidence that joint immobility alone causes joint contracture. The absence of significant changes in soleus muscle outcomes should also be interpreted cautiously. This rat model provides a proof-of-concept that may inform future studies of intermittent joint immobility in humans.
